# Interesting Analyses of Articles in Foreign Journals

**Published:** 1864-07

**Authors:** 


### INTERESTING ANALYSES OF ARTICLES IN FOREIGN JOURNALS.

### *Surgical Experience at St. Bartholomew's.* By FREDERICK SKEY, Esq., F. R. S.

*Bursæ.*

The bursa in front of the knee joint is situated partly on the lower half of the patella, and partly on the tendon of the quadriceps muscle, commonly called the ligamentum patellæ. When diseased, it exhibits the following characters: It may be small or large in size, varying from that of the half of a small apple to the size and form of a large orange. The swelling may be hard or soft, composed of thick walls of lymph, with a small central cavity, forming a mere cleft or fissure, or more commonly consisting of the bursa dilated by a collection of serum or pus. It may be active and painful, or chronic and painless. In both cases it is an evil and an inconvenience, and demands the best resources of surgery for its cure. The subjects of this disease, on applying for hospital relief, state themselves to have been under treatment for weeks or even months. The treatment in question resolves itself in two varieties: 1st, by the repeated application of blisters; 2d, by that of tincture of iodine. Both are inoperative, for at the expiration of many weeks the disease triumphs. All the above varieties of bursal disease are readily amenable to the influence of a moderately thick silk thread passed through them. The effect of the thread is the destruction of the bursa, whether composed of solid walls or fluid contents, and the formation of an abscess. The period required for this conversion varies from three to ten or twelve days. The presence or the immediate advent of matter is indicated by pain in the swelling and by the existence of a red halo around the openings made by the needle. When this sign is *fully* established, the thread may be withdrawn. The bursa is now and forever obliterated, and we have an abscess in its place, identical with, and amenable to the same treatment as an abscess in any other place.

*Burns.*

In the treatment of burns I have had some experience. Every year confirms my conviction of the soundness of the principle first proclaimed to our profession by Dr. Kentish, of Bristol, about half a century ago—a principle approved, accepted, and all but universally adopted for twenty years and upwards. That principle consisted in the application of stimulants in one or other form as early as possible after the receipt of the injury. Although less popular in 1863, it is not less sound. How many hospital surgeons have subjected these painful accidents to the test of critical inquiry and experiment? How many can truly affirm, "I have fairly tried the two methods of treatment, and I adopt the Carron-oil system? Of the two systems of treatment which consist of medical or other agents, the one adopts the principle of stimulation, the other soothes. The idea of soothing an irritated surface is indeed plausible. To soothe signifies to allay, to calm. What is the agent employed for this purpose—almost universally employed? Carron oil. Ask a man the subject of a burn what amount of relief from suffering he obtains from the application of Carron oil at the expiration of any term from one hour to forty-eight. I believe it neither affords relief, nor answers any useful purpose whatever. Its employment is little better than a delusion. Yet it is the almost universal agent used in the coal and iron districts, in which injuries by fire are so frequent and so fatal. The reason why it is so universally employed in these districts may be inferred from the following anecdote: Some years since, while on a visit near the iron works of Dowlais and Merthyr Tydvil, where many thousand men were engaged, I witnessed sundry cases of severe burns; and on my urging on the resident surgeon the value of the opposite principle to that which he adopted in the treatment, he replied to this effect: "If I were to attempt to invade the prejudices of these men, and insist on a change of treatment, my life would hardly be safe from their violence." This is indeed valuable testimony. A very common fact in every-day life displays the value of the treatment by stimulants. The smarting pain caused by a burn or scald on the hand is relieved, and, if not very severe, is almost removed, by subjecting the hand to the influence of the heat of a fire. The closer it is held—the more severe the pain caused by the undue heat, the greater is the amount of permanent relief. What is the theory of this? I cannot pretend to give a satisfactory explanation of the phenomenon, but it is not the less true. It is palpable to ordinary observation that if we stimulate a burnt region of the body, or, in other words, if we increase the pain of the part by the application of any agent—as heat, whether from fire or hot water; or if we stimulate by the agency of turpentine, as recommended by Dr. Kentish, or spirit or stimulant of any description—we relieve the affected part from its pain, and carry it forwards by a rapid stride towards recovery. The greater pain deadens the lesser, and not for the hour merely, but permanently. And what is true in principle on the smaller scale is equally true on the larger. Of stimulants, I know no agent so efficient as a solution of nitrate of silver, which I have for some years employed in the proportion of from ten to fifteen grains to an ounce of water for an adult, and from five to seven grains for a child. This solution applied freely over the burnt surface is followed by the application of cotton wool. In an hour or less the pain decreases, and ere long subsides. I could illustrate the efficacy of this treatment by an endless number of examples, but I will mention one only. Five men were severely burnt by an explosion of gas, and were brought to the hospital. One died immediately: the remaining four were badly burnt about the face, chest and arms. The face and chest of each man was washed with a solution of ten grains of nitrate of silver; to the arms the celebrated Carron or boiled oil was applied. Twenty-four hours elapsed, and on inquiry whether the patients were suffering pain, each made the same reply: "I am easy everywhere, except in the arms and hands." The oil was removed, the solution was applied, and relief followed immediately. The solution may be profitably applied at any period, so long as the pain remains.

On the younger members of our profession I most strongly urge the value of the stimulating principle in the treatment of burns and scalds of every description, not only for the relief from suffering which it affords, but for the influence it exerts in abridging the duration of the consequences of the injury and promoting an early recovery. The only exceptions to this benefit are found in the cases of very severe injury which are attended by great destruction of the tissues of the affected parts; but even in such cases relief from pain is always afforded by the application of the solution in or about the strength I have mentioned.

---

**PERSEVERANCE.—The chair of Surgery of the Faculty of Turin was lately competed for by Messrs. Pacchiotti and Bottini. The former beat his adversary and obtained the professorship, for which he had competed *five times* previously.**

*On Vaginal Lithotomy.*

Dr. Aveling read before the Obstetrical Society of London a paper reciting the particulars of thirty-five cases in which this operation had been performed—twelve British and twenty-two foreign. The author also gave another case, in which he divided the vesico-vaginal septum, and extracted a small rough stone. The wound was brought together with silver-wire sutures. Gilt beads were passed over the ends of these and run down to the lips of the wound. These were kept in position by a perforated shot, also passed over the end of the sutures and tightened upon them by a pair of forceps. He proposes in future to use a coil, made by winding a piece of the suture-wire round a pin, instead of the beads. The wound healed in a week, and the patient returned to her home in a fortnight.

Mr. Spencer Wells congratulated Dr. Aveling upon the successful result of his interesting case, and heartily concurred in the tribute he had paid to Dr. Marion Sims. But he (Mr. Wells) had begun to doubt whether the success which had followed the operation for vesico-vaginal fistula of late years was so much due to the use of wire sutures as to the improvements which Dr. Sims had originated in the mode of bringing the fistula into view, accurately paring the edges, and bringing them into perfect opposition. Provided the edges of a fistula were thoroughly pared and kept in close apposition, it was probably of little importance how this was done. A year ago he (Mr. Wells) was as strongly in favor of metallic sutures as anybody, but latterly a wider experience had taught him that it is only after five or six days that wires show any advantage over silk, and before that time the sutures ought to be removed. Then silk offers the great advantages over wire of being more easily applied, of not requiring so large a needle to pass it, of the ends being much less irritating, and of being more easily removed. After many comparative trials of different parts of the same wound with wires of silver, iron, lead, platinum, and aluminum, with fine catgut, horsehair, telegraph wire, india-rubber thread, and and the fine strong silk known as "Chinese twist," he had become convinced that wires offered no advantage over silk, while silk offered many advantages over every other material used for sutures. In a recent case he had closed a vesico-vaginal fistula by five silk sutures, and perfect union resulted, although no catheter was used. The supposed necessity for the use of the catheter after closing vaginal fistula was another error which time was correcting. The urine is by no means so irritating a fluid as some believe. The lower orders use it as a lotion to the eyes and to sore legs, and it certainly cannot differ much from the dilute saline solutions constantly prescribed as astringents or stimulants. The use of the catheter is the most troublesome part of the after-treatment, and often most distressing to the patient. One of his patients really could not bear it, yet she did perfectly well; and lately he had not used it at all, union taking place quite as well as when it was used, and the patient being much more comfortable. With regard to stone in the bladder during labor being a cause of vesico-vaginal fistula, he had once removed in the Samaritan Hospital a larger stone through a fistula before closing it, but it was very questionable whether it could often be necessary to remove a calculus through the vagina when no fistula existed, or to run the risk of making a fistula to remove a stone. Lithotrity was very easily performed in women; and large fragments of stone passed readily through the short female urethra, so that no form of lithotomy could often be called for. Simple dilatation of the urethra was not likely to answer in any case not suitable for lithotrity, and its effects are very uncertain—a large stone might be removed and no incontinence follow, but incontinence might follow removal of a very small calculus. The usual aid to dilatation by incising the urethra was still worse. A surgeon of very large experience had told him that he had done it for two adults and seven children, and "they were all drebblers." Where from some exceptional condition of bladder and stone, lithotrity was inappropriate, vaginal lithotomy might, therefore, become a valuable operation; but experience was still wanting to show that it was better than, or as good as, the lateral operation so successfully practised by Dr. Buchanan, of Glasgow.

---

*Relative Rank of Medical Officers in the British Service.*

Her Majesty has been graciously pleased to direct, by her order in Council, dated April 16th, 1861, that the relative rank of Medical Officers in the Navy and Army shall, in future, be as follows:

1. Inspector-General of Hospitals and Fleets, after three years' service, on full pay as such, to rank with Major General, according to the date of the completion of the said three years' service.

2. Inspector General of Hospitals and Fleets, under three years' service, on full pay as such, to rank as Brigadier-General, according to the date of commission.

3. Deputy Inspector-General of Hospitals and Fleets, after five years service, on full pay as such, to rank with Colonel, according to the date of the completion of the said five years' service.

4. Deputy Inspector-General of Hospitals and Fleets, under five years service, on full pay as such, to rank as Lieutenant-Colonel, according to the date of the commission.

5. Staff-Surgeon, to rank with Lieutenant-Colonel, but junior of that rank.

6. Surgeon, to rank with Major, according to the date of commission.

7. Assistant-Surgeon, after six years' service, on full pay as such, to rank with Captain, according to the date of the completion of the said six years' service.

8. Assistant-Surgeon, under six years' service, on full pay as such, to rank with Lieutenant, according to the date of commission.

By command of their Lordships,

C. Paget.

---

*Contents.*

